# Immune heterogeneity and therapeutic resistance in gynecological malignancies

**DOI:** 10.3389/fimmu.2026.1837428

**Published:** 2026-06-22

**Authors:** Mengyi Zhang, Binhan Guo

**Affiliations:** 1 Department of Laboratory Medicine, West China Second University Hospital, Sichuan University, Chengdu, China; 2 Key Laboratory of Birth Defects and Related Diseases of Women and Children (Sichuan University), Ministry of Education, Chengdu, China

**Keywords:** drug resistance, gynecological malignancies, NK cell, single-cell sequencing, T cell, tumor heterogeneity, tumor microenvironment

## Abstract

Gynecological malignancies are characterized by profound cellular heterogeneity, dynamic immune remodeling, and frequent therapeutic resistance, which together limit durable clinical responses. Single-cell sequencing has emerged as a powerful approach for dissecting tumor ecosystems at unprecedented resolution, enabling the identification of malignant subclones, immune cell states, stromal subsets, lineage trajectories, and intercellular communication networks. In cervical cancer, single-cell studies have revealed epithelial diversity, HPV-associated immune suppression, exhausted T/NK-cell populations, macrophage polarization, and genomic alterations linked to chemoradiotherapy resistance. In ovarian cancer, single-cell analyses have refined the understanding of fallopian tube origin, molecular subtypes, metastatic dissemination, ascites-associated immune suppression, platinum resistance, and tertiary lymphoid structure-related immune phenotypes. In endometrial cancer, single-cell profiling has uncovered heterogeneous cancer-associated fibroblast populations, epithelial biomarkers, and stromal–immune interactions with prognostic relevance. This review summarizes recent advances in single-cell sequencing across cervical, ovarian, and endometrial cancers, with particular emphasis on immune heterogeneity, tumor–microenvironment crosstalk, therapeutic resistance, and emerging opportunities for precision immunotherapy.

## 1 Introduction

Single-cell sequencing has revolutionized biomedical research by enabling high-resolution dissection of transcriptomes, genomes, proteomes, epigenomes, and spatial architectures (1–3). This technological advance provides unprecedented capacity to decipher complex biological systems, particularly in oncology, where cellular diversity underpins disease pathogenesis and therapeutic response. Gynecological malignancies remain a leading cause of cancer-related mortality among women globally (4), comprising intricate ecosystems of malignant, immune, stromal, and endothelial subpopulations with divergent biological behaviors (5). Critically, interpatient heterogeneity—even among tumors sharing stage, anatomical site, and histology—underlies substantial variability in therapeutic responses and clinical outcomes (6, 7).

Conventional bulk sequencing, while informative, yields population-averaged signals that obscure cell-intrinsic variation and mask rare but clinically relevant subclones (8). By contrast, single-cell approaches resolve genomic, transcriptomic, proteomic, and epigenetic features at individual-cell resolution, thereby delineating the composition, functional states, and intercellular crosstalk within heterogeneous tumor ecosystems (9). This capability enables precise identification of malignant and microenvironmental subsets driving tumor initiation, progression, metastasis, and therapeutic resistance, positioning single-cell sequencing as a pivotal tool for advancing precision oncology (10). Here, we summarize recent applications of single-cell technologies across cervical, ovarian, and endometrial cancers, emphasizing their utility in decoding immune heterogeneity, elucidating resistance mechanisms, and informing novel immunotherapeutic strategies for improved patient stratification and personalized treatment.

## 2 Immune microenvironment in gynecological malignancies

The tumor immune microenvironment of gynecological malignancies is characterized by profound cellular heterogeneity and dynamic immunometabolic interactions that collectively regulate tumor progression, metastatic dissemination, immune escape and therapeutic resistance (11–13). Single-cell sequencing technologies have substantially improved the understanding of how immune and stromal populations interact with malignant cells through cytokine networks, metabolic competition and inflammatory signaling cascades (14, 15). In cervical cancer, ovarian cancer and endometrial cancer, the TIME consists of diverse innate and adaptive immune cell populations that exhibit context-dependent pro-tumorigenic or anti-tumorigenic functions (11, 13, 16, 17).

### 2.1 Innate immune cells and inflammatory remodeling

Innate immune cells constitute the first line of immune surveillance within gynecological tumors and are deeply involved in shaping inflammatory and immunosuppressive microenvironments (18). Tumor-associated macrophages (TAMs), particularly M2-like macrophages, are among the most abundant immune populations in cervical cancer, ovarian cancer and endometrial cancer. Single-cell studies have demonstrated that TAMs highly express CD163, CD206, IL10 and TGF-β, thereby promoting immunosuppression, extracellular matrix remodeling and angiogenesis (19, 20). In cervical cancer, HPV-induced chronic inflammation activates NF-κB and STAT3 signaling, which enhances CCL2-mediated monocyte recruitment and drives macrophage polarization toward the M2 phenotype (21, 22). M2-like TAMs further secrete VEGF, CXCL8 and matrix metalloproteinases (MMPs), thereby facilitating tumor angiogenesis and invasion (23, 24). Moreover, lactate accumulation within hypoxic tumor regions promotes HIF-1α signaling in TAMs, driving glycolytic metabolic reprogramming and reinforcing immunosuppressive phenotypes through increased ARG1 and PD-L1 expression (25, 26). In ovarian cancer, ascites-derived TAMs exhibit strong immunoregulatory activity mediated through IL-6/JAK/STAT3 and CSF1 signaling pathways (27–29). These macrophages suppress cytotoxic T-cell activity while promoting epithelial–mesenchymal transition (EMT) and platinum resistance (30). Single-cell analyses further revealed that TAMs participate in lipid metabolic reprogramming by transferring fatty acids to tumor cells through fatty acid-mediated mechanisms, thereby supporting metastatic colonization and oxidative phosphorylation (31, 32). Additionally, TAM-derived TGF-β activates CAFs and contributes to extracellular matrix stiffness and fibrotic remodeling (32, 33). In endometrial cancer, TAM infiltration is associated with PI3K/AKT/mTOR activation and enhanced expression of inflammatory cytokines such as TNF-α and IL-1β, which promote tumor proliferation and immune evasion (34–36).

Cancer-associated fibroblasts (CAFs) also play central roles in remodeling the inflammatory microenvironment. Distinct CAF subtypes, including inflammatory CAFs (iCAFs), antigen-presenting CAFs (apCAFs), and vascular CAFs (vCAF), have been identified in endometrial and ovarian cancers (37–39). CAF-derived CXCL12 interacts with CXCR4 on tumor cells and immune cells to promote immune exclusion and metastatic dissemination (38, 40). Furthermore, CAF-secreted TGF-β suppresses NK-cell cytotoxicity and induces Treg-cell differentiation. Metabolically, CAFs undergo aerobic glycolysis and produce lactate and pyruvate, which are utilized by tumor cells to sustain oxidative metabolism and therapeutic resistance (41, 42). Natural killer (NK) cells are critical mediators of anti-tumor innate immunity; however, their function is frequently impaired in gynecological malignancies (43–45). In cervical cancer and ovarian cancer, TGF-β and IL-10 suppress NK-cell activation through SMAD-dependent pathways, leading to reduced expression of perforin, granzyme B and NKG2D. Tumor-derived exosomal miRNAs and lactate accumulation additionally impair NK-cell mitochondrial metabolism and cytotoxic function (46–48). Single-cell analyses have identified exhausted NK-cell subsets expressing TIGIT, PD-1 and LAG3, suggesting profound functional dysfunction within advanced tumors (49, 50).

Dendritic cells (DCs) are essential antigen-presenting cells linking innate and adaptive immunity. However, tumor-associated DCs frequently exhibit tolerogenic phenotypes characterized by low CD80/CD86 expression and impaired antigen presentation. VEGF, IL-6 and prostaglandin E2 suppress DC maturation through STAT3 activation, thereby limiting CD8^+^ T-cell priming (51–53). In ovarian cancer ascites, plasmacytoid DCs highly express IDO1 and contribute to Treg-cell expansion through kynurenine-mediated immunosuppression (54–56). Neutrophils and eosinophils also contribute to inflammatory remodeling in gynecological malignancies. Tumor-associated neutrophils (TANs) promote metastasis through neutrophil extracellular trap (NET) formation and CXCL8-mediated recruitment (57, 58). Activation of PI3Kγ and NF-κB signaling further enhances neutrophil-mediated angiogenesis and immunosuppression (59). In contrast, eosinophils may exert dual functions depending on the cytokine milieu. IL-5 and eotaxin signaling can recruit eosinophils to tumors, where they release cytotoxic granules and inflammatory mediators (60, 61); however, persistent eosinophilic inflammation may also enhance tissue remodeling and tumor progression through TGF-β secretion.

### 2.2 Adaptive immune cells and immunometabolic reprogramming

**Figure 1:**
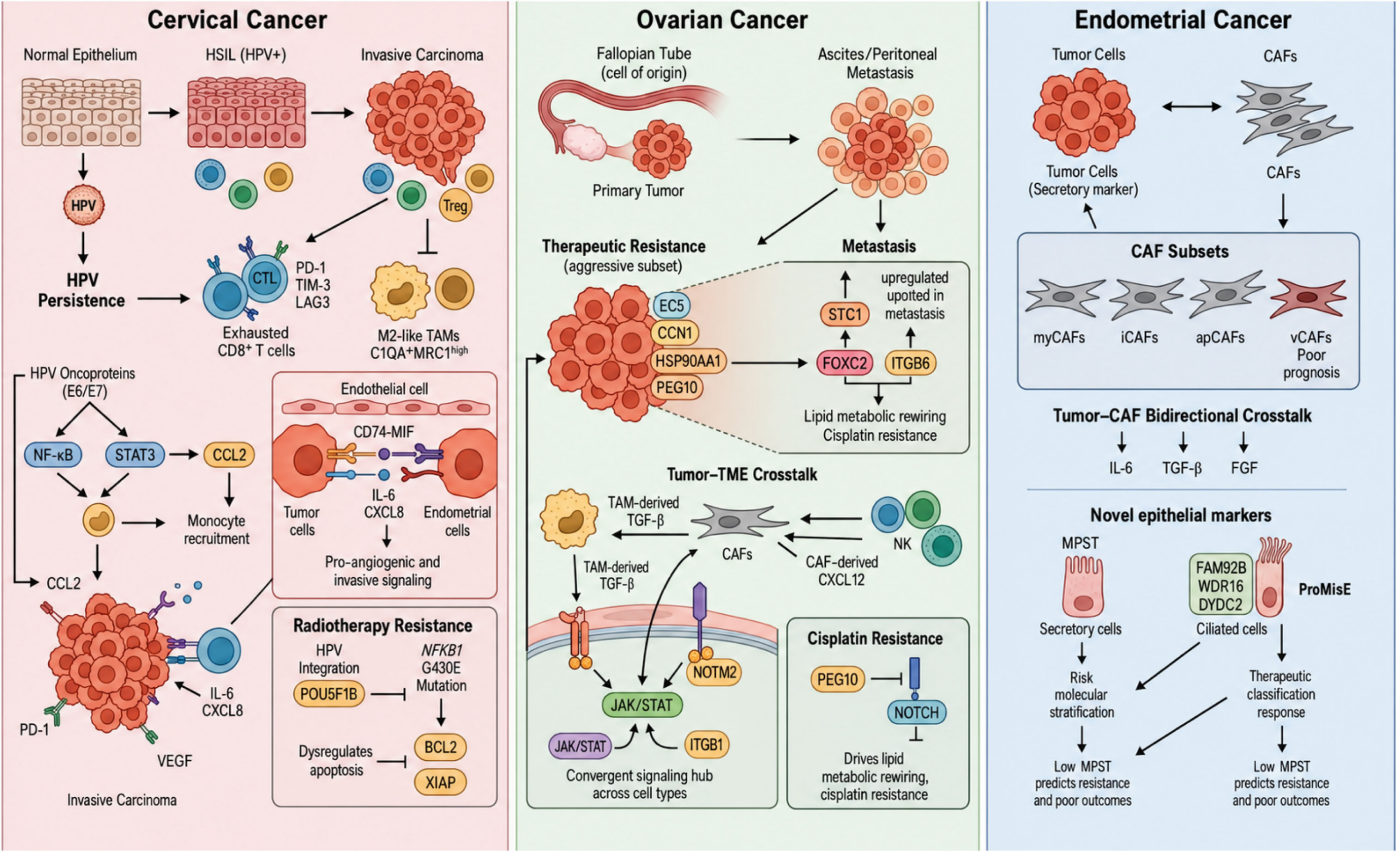
Immune heterogeneity in gynecological malignancies.

Adaptive immune cells are central regulators of anti-tumor immunity in gynecological malignancies. CD8^+^ cytotoxic T lymphocytes (CTLs) represent the major effector population responsible for tumor-cell elimination (62, 63). Nevertheless, chronic antigen stimulation, hypoxia and metabolic stress drive T-cell exhaustion characterized by elevated PD-1, TIM-3, TIGIT and LAG3 expression. In cervical cancer, persistent HPV antigen exposure activates PD-1/PD-L1 and CTLA-4 signaling, thereby impairing T-cell receptor (TCR) signaling and cytokine production (64, 65). Single-cell analyses demonstrated that exhausted CD8^+^ T cells exhibit impaired mitochondrial oxidative phosphorylation and increased dependence on glycolysis, accompanied by reduced IFN-γ and TNF-α secretion (66, 67). In ovarian cancer, the immunosuppressive ascitic microenvironment profoundly reshapes T-cell metabolism. Elevated lactate, adenosine and reactive oxygen species inhibit T-cell proliferation and mitochondrial respiration (68–70). Tumor cells compete with T cells for glucose and glutamine through enhanced aerobic glycolysis and glutaminolysis, thereby restricting nutrient availability for effector lymphocytes (71, 72). Activation of the PI3K/AKT/mTOR pathway in tumor cells further enhances glucose uptake and suppresses T-cell functionality (73, 74). Furthermore, CXCL9/CXCL10-CXCR3 signaling regulates T-cell trafficking into tumors, while chronic IFN-γ exposure induces adaptive PD-L1 upregulation in malignant cells (75–77). B cells also contribute to adaptive immune remodeling through cytokine secretion, antibody production and antigen presentation (78). However, regulatory B cells (Bregs) can suppress anti-tumor immunity by producing IL-10 and TGF-β, thereby inhibiting CD8^+^ T-cell responses and promoting Treg expansion (79). Single-cell analyses have further revealed tertiary lymphoid structure-associated B-cell populations linked to improved prognosis in subsets of ovarian cancer patients (**Figure 1**) (80, 81).

## 3 Single-cell sequencing in gynecological malignancies

The tumor immune microenvironment, including the influence of immune cells and signaling regulatory pathways, plays a role in gynecological malignancies, and although various immune checkpoint inhibitors, cellular immunotherapies, and immune adjuvants have been proposed and evaluated in clinical trials, only a small subset of patients have benefited from these treatments (82–84). Differences in individual tumor microenvironments may be a key factor. In recent years, the development of single-cell technologies has provided unprecedented prospects for precision immunotherapy (38, 85, 86).

### 3.1 Cervical cancer

#### 3.1.1 Tumor heterogeneity and T immune dynamics during cervical cancer exploration

Tumor heterogeneity is a defining feature of cancer and underlies differences in histology, immune contexture, proliferative capacity, invasion, metastasis and therapeutic response (87, 88). Cervical cancer cells were analyzed and resolved four major epithelial clusters with distinct functional identities (89). Cervical cancer progresses from HPV infection through intraepithelial neoplasia to invasive carcinoma, yet the immune mechanisms governing this trajectory remain incompletely defined (90, 91). Single-cell transcriptomics has enabled high-resolution mapping of immune remodeling across disease stages. Analysis of normal cervix, high-grade squamous intraepithelial lesions (HSIL), primary tumors, and metastatic lymph nodes revealed T/NK cells as predominant immune constituents (50). Immune profiling identified stage-specific CD8^+^ T-cell states: HSIL harbored tissue-resident memory (TRM-like) subsets indicative of limited activation; primary tumors exhibited exhausted CD8^+^ T cells reflecting immunosuppression; whereas metastatic nodes were enriched for cytotoxic and effector-memory CD8^+^ T cells, suggesting renewed immune engagement (49, 92). Subsequent scRNA-seq of primary tumors and lymph nodes further demonstrated that metastatic lesions harbor tumor cells with elevated cell-cycle and immune-regulatory signaling, alongside increased CD206 expression (93). C1QA^+^MRC1^high^ macrophages and antigen-presenting cancer-associated fibroblasts in metastatic niches may facilitate immune evasion and dissemination (93, 94). These findings delineate dynamic immune adaptations during cervical cancer progression and inform stage-targeted therapeutic strategies.

#### 3.1.2 Tumor microenvironment description

The tumor microenvironment in cervical cancer represents a dynamically coordinated ecosystem wherein malignant epithelial cells co-evolve with stromal, immune, and endothelial compartments through intricate intercellular crosstalk (49, 95). Single-cell transcriptomic profiling has systematically delineated this cellular architecture, identifying fibroblasts, endothelial cells, T/NK cells, macrophages, neutrophils, B cells, and mast cells as functionally integrated components that collectively drive tumor progression via ligand–receptor signaling networks (89). Notably, tumor–endothelial communication in malignant tissues is predominantly mediated by immune-regulatory axes—including CD74-COPA, CD74-MIF, and CD74-APP—whereas normal epithelia preferentially express structural and differentiation-related pairs such as DSG2-DSC3 and EGFR-TGFB1. Intriguingly, the convergence of ligand–receptor expression between tumor and immune cells suggests synergistic cooperation in orchestrating pathological angiogenesis (96, 97). The CD74-MIF axis functions as a central signaling hub: MIF binding activates CD74 on endothelial and immune cells, triggering NF-κB, PI3K/AKT, and MAPK cascades that enhance tumor-cell survival, proliferation, and apoptosis resistance (22, 98). Concurrently, CD74-MIF signaling induces secretion of pro-inflammatory and pro-angiogenic mediators (IL-6, CXCL8, VEGF), thereby promoting endothelial activation, vascular remodeling, and invasive potential (99–101). Furthermore, this pathway reinforces immunosuppression by recruiting macrophages, fostering M2-like polarization, and impairing cytotoxic T-cell function (102, 103). Hence, CD74-centered ligand–receptor interactions establish a mechanistic nexus linking tumor–immune–endothelial crosstalk to angiogenic remodeling, immune evasion, and disease progression in cervical cancer.

#### 3.1.3 Evaluating treatment response

Therapeutic resistance to radiotherapy and chemotherapy constitutes a principal determinant of suboptimal outcomes in cervical cancer. Single-cell sequencing provides unprecedented resolution to dissect the molecular architecture of treatment failure and refine response stratification (104, 105). Researchers applied single-cell transcriptomics to cells from four cervical cancer specimens, delineating nine distinct cellular populations; pathway enrichment analysis revealed that differentially expressed genes between chemoresistant and chemosensitive patients were predominantly associated with PI3K/AKT and MAPK signaling, implicating these cascades in therapeutic evasion (106). Complementarily, single-cell whole-genome sequencing on pre- and post-radiation tumor cells from an HPV-positive patient demonstrates the dynamic clonal selection wherein minor subpopulations expanded following treatment (107). Post-radiation tumor cells harbored HPV integration within POU5F1B and exhibited increased frequency of NFKB1 G430E missense mutations. Functional validation indicated that NFKB1 alterations compromise tumor-suppressive activity and diminish radiosensitivity, positioning NFKB1 as a promising target to augment radiotherapeutic efficacy (108, 109). HPV integration may destabilize host genomic architecture and dysregulate adjacent oncogenic or stemness-associated loci, fostering clonal resilience under therapeutic pressure (110, 111). Concurrently, NFKB1 dysfunction perturbs NF-κB–mediated transcriptional control of apoptosis-related and stress-response genes, including BCL2, XIAP and survivin, thereby attenuating radiation-induced cytotoxicity (112). Furthermore, radiation-triggered DNA damage activates repair pathways, enabling subclones with HPV integration or NF-κB pathway alterations to evade apoptosis and persist post-treatment (113, 114). These single-cell insights elucidate how genomic instability and signaling dysregulation converge to drive therapeutic resistance, providing a rational foundation for mechanism-informed intervention strategies in cervical cancer.

### 3.2 Ovarian cancer

#### 3.2.1 Exploration of tumor origin and molecular subtyping

High-grade serous ovarian cancer (HGSOC), the most lethal gynecological malignancy (115, 116), is increasingly recognized to originate from fallopian tube epithelium, a paradigm robustly supported by single-cell transcriptomic evidence endorsing the dualistic model (117, 118). Smart-seq2 has been employed to delineate molecular subtypes within normal fallopian tube epithelium; projection of these signatures onto malignant cells substantiated tubal origin and identified an epithelial–mesenchymal transition–high (EMT-high) subtype associated with adverse prognosis (119). Concurrently, single-cell multi-omics analysis revealed that chromosomal amplifications, particularly those encompassing MYC, drive ovarian cancer through proto-oncogene dosage effects, underscoring copy-number alterations as pivotal tumorigenic events (120). Beyond cell-of-origin insights, single-cell sequencing has refined molecular stratification by resolving intratumoral heterogeneity (121, 122). Epithelial cells were classified from primary and metastatic HGSOC into five functional subpopulations: EC5, enriched for proliferative, DNA damage-response, and chemoresistance programs, denotes an aggressive state, whereas EC2, expressing ciliated epithelial markers (FOXJ1, PIGR, CAPS, GDF15), reinforces tubal derivation (123). Profiling malignant ascites from HGSOC patients, 18 subpopulations were identified to predominantly reflect stromal and immune compartment composition, particularly cancer-associated fibroblasts and macrophages, rather than intrinsic tumor-cell states (124). Notably, convergent JAK/STAT pathway enrichment across malignant and non-malignant compartments emerged as a druggable vulnerability, with pharmacological inhibition significantly impairing HGSOC viability (125). Collectively, these single-cell discoveries reframe HGSOC classification, emphasizing ecosystem-level interactions and actionable signaling nodes for precision therapeutic development.

#### 3.2.2 Tumor metastasis and drug resistance

Single-cell sequencing has elucidated the molecular architecture underlying ovarian cancer metastasis and therapeutic resistance (126, 127). Peritoneal dissemination and lymphatic spread represent predominant metastatic routes; notably, integrated genomic and transcriptomic profiling has demonstrated that genetically clonal tumor cells undergo minimal transcriptional reprogramming during peritoneal metastasis, whereas lymph node colonization involves extensive differential gene expression, implying early acquisition of peritoneal metastatic fitness (120). This line of investigation further identified CCN1 and HSP90AA1 as metastasis-associated drivers whose genetic or pharmacological inhibition attenuates invasive capacity (120). Complementarily, multi-regional single-cell analyses have revealed progressive STC1 upregulation from primary to metastatic lesions, with functional validation confirming its role in promoting metastasis, lipid metabolic rewiring, and cisplatin resistance via the FOXC2/ITGB6 axis (125). A distinct PEG10^+^ embryonic-like epithelial subset has been characterized as sustaining stemness and conferring cisplatin resistance through NOTCH signaling activation (128). Furthermore, integrated single-cell transcriptomics with TCR/BCR profiling in platinum-resistant recurrent HGSOC has identified a proliferative EC3 subpopulation exhibiting enriched chemoresistance-associated transcripts, G2/M-phase predominance, and heightened metabolic activity (129). Concurrently, chemotherapy-induced T-cell senescence and altered TCR/BCR clonality underscore dynamic immune remodeling accompanying therapeutic failure (129). Beyond epithelial heterogeneity, single-cell analyses reveal an immunosuppressive microenvironment in HGSOC, wherein exhausted CD8^+^ T cells (PD-1^+^TIM-3^+^TIGIT^+^) and FOXP3^+^ regulatory T cells collectively impair anti-tumor immunity through checkpoint signaling, cytokine-mediated suppression, and metabolic competition (38, 85, 130). These integrated insights highlight metastasis- and resistance-associated cellular programs as promising targets for precision intervention in ovarian cancer.

### 3.3 Endometrial cancer

Endometrial cancer exhibits profound tumor heterogeneity, encompassing not only genetic and phenotypic diversity among malignant epithelial cells but also substantial variation in fibroblasts, immune infiltrates, and stromal constituents within the tumor microenvironment (131, 132). Cancer-associated fibroblasts (CAFs), the predominant stromal population, function as critical orchestrators of tumor progression, metastasis, and therapeutic resistance (133, 134). Single-cell transcriptomic analyses have resolved CAFs into four functionally distinct subpopulations—stromal, inflammatory, antigen-presenting, and vascular CAFs—among which vascular CAFs correlate with inferior survival outcomes and represent promising therapeutic targets (135). Ligand–receptor interaction mapping further elucidates extensive bidirectional crosstalk between malignant epithelial cells and CAF subsets, cooperatively driving endometrial cancer progression through pro-angiogenic and immunomodulatory signaling cascades. In parallel, single-cell profiling of normal endometrium has facilitated the discovery of novel cell-type-specific markers, including MPST for secretory epithelium and FAM92B, WDR16, and DYDC2 for ciliated epithelium (136). These markers are retained in endometrial malignancies and exhibit significant prognostic relevance; notably, MPST expression enables refined risk stratification within the established ProMisE molecular classification framework, thereby enhancing prognostic precision and supporting personalized therapeutic decision-making (137–139). Overall, these single-cell insights advance biomarker discovery and deepen mechanistic understanding of stromal–epithelial interactions underlying endometrial carcinogenesis.

## 4 Conclusion

Single-cell sequencing has substantially advanced the understanding of gynecological malignancies by resolving the complex cellular ecosystems that are obscured by conventional bulk profiling. Across cervical, ovarian, and endometrial cancers, single-cell studies have revealed that tumor progression and treatment failure are not driven solely by malignant epithelial cells, but instead arise from dynamic interactions among tumor cells, immune cells, stromal components, endothelial cells, and metabolic niches. In cervical cancer, HPV-driven inflammation, T-cell exhaustion, macrophage polarization, ligand–receptor signaling, and therapy-selected resistant clones collectively shape disease evolution. In ovarian cancer, single-cell analyses have clarified tumor origin, metastatic fitness, ascites-associated immune suppression, chemoresistant epithelial states, and immune remodeling during recurrence. In endometrial cancer, the identification of distinct CAF subsets and epithelial markers has provided new insights into stromal–epithelial communication, prognostic stratification, and potential therapeutic vulnerabilities.

Despite these advances, several challenges remain before single-cell technologies can be fully integrated into clinical practice. Current studies are often limited by small sample sizes, tumor sampling bias, batch effects, incomplete spatial information, and insufficient prospective validation. Future research should integrate single-cell transcriptomics with spatial omics, epigenomics, proteomics, TCR/BCR sequencing, and longitudinal clinical data to reconstruct tumor evolution and immune escape more comprehensively. In parallel, functionally validated biomarkers and resistance-associated cell states should be translated into clinically applicable tools for patient stratification, therapeutic monitoring, and rational combination therapy. Ultimately, single-cell sequencing provides a transformative framework for decoding immune heterogeneity and therapeutic resistance in gynecological malignancies, offering new opportunities to develop more precise, individualized, and durable treatment strategies.
